# Supplementary material for: Resolving the full spectrum of human genome variation using Linked-Reads
Source: Genome Res. 2019 Apr;29(4):635–45. doi: 10.1101/gr.234443.118 (PMC6442396; doi:10.1101/gr.234443.118)
Supplement: Supplemental Material [file supp_gr.234443.118_Supplemental_longranger-2.2.zip › longranger-2.2.tar.bz2!/lr2.2-public/lib/stan/cmdstan/stan/doc/api/html/structstan_1_1gm_1_1validate__assignment_1_1result.html]

Stan: stan::gm::validate\_assignment::result< T1, T2, T3, T4 > Struct Template Reference


|  |  |
| --- | --- |
|  | Stan  2.5.0  probability, sampling & optimization |


- Main Page
- Related Pages
- Namespaces
- Classes
- Files

- Class List
- Class Index
- Class Hierarchy
- Class Members

All Classes Namespaces Files Functions Variables Typedefs Enumerations Enumerator Friends Macros Pages

- stan
- gm
- validate\_assignment
- result

Public Types |
List of all members

stan::gm::validate\_assignment::result< T1, T2, T3, T4 > Struct Template Reference

`#include <statement_grammar_def.hpp>`

|  |  |
| --- | --- |
| Public Types | |
| typedef bool | type |
|  | |

## Detailed Description

### template<typename T1, typename T2, typename T3, typename T4> struct stan::gm::validate\_assignment::result< T1, T2, T3, T4 >

Definition at line 126 of file statement\_grammar\_def.hpp.

## Member Typedef Documentation

template<typename T1 , typename T2 , typename T3 , typename T4 >

typedef bool stan::gm::validate\_assignment::result< T1, T2, T3, T4 >::type

Definition at line 126 of file statement\_grammar\_def.hpp.

---

The documentation for this struct was generated from the following file:

- src/stan/gm/grammars/statement\_grammar\_def.hpp


---

[ Stan Home Page ]

*© 2011–2014,
Stan Development Team.*
